# Impact of environmental pollution on acne: a systematic review

**DOI:** 10.1093/skinhd/vzaf090

**Published:** 2025-12-30

**Authors:** Isobel R Okeah, Usamah M Afzal, Faisal R Ali

**Affiliations:** School of Medicine and Dentistry, University of Lancashire, Preston, UK; Department of Dermatology, Harrogate District Hospital, Harrogate, UK; School of Medicine and Dentistry, University of Lancashire, Preston, UK

## Abstract

In an increasingly urbanized world, environmental pollution is recognized for its adverse effects on both systemic and skin health. While its role in conditions such as atopic dermatitis and psoriasis is well documented, its impact on acne vulgaris remains less clear. This review aims to evaluate existing literature examining the association between environmental pollutants – such as particulate matter (PM_2.5_, PM_10_), nitrogen oxides (NO_2_, NO_x_) and traffic-related emissions – and the development, severity or exacerbation of acne. A systematic search of peer-reviewed English-language studies published between 2010 and 2025 was conducted using PubMed. Search terms included ‘air pollution’, ‘particulate matter’, ‘PM_2.5_’, ‘PM_10_’, ‘NO_2_’, ‘NO_x_’, ‘environmental pollution’, ‘traffic pollution’, ‘acne’ and ‘acne vulgaris’. Studies were included if they investigated the relationship between environmental pollutants and acne in human populations. Of the 27 studies identified, 17 met inclusion criteria. Systematic reviews were also incorporated to provide broader context. Several studies demonstrated significant associations between pollutant exposure and acne exacerbation. A time-series study in China involving 71 625 outpatient visits found that each 10 μg m^–3^ increase in SO_2_ and NO_2_ correlated with 1.02% and 2.13% increases in acne-related visits, respectively. Other studies appear to show pollutants being associated with increased sebum production and reduction of antioxidants. Proposed mechanisms include oxidative stress, microbiome disruption and follicular hyperkeratinization. However, study heterogeneity, lack of diversity and limited control for confounders limit generalizability. Longitudinal research is needed to clarify pollution’s role in acne and inform targeted prevention strategies.

## Teaser

This systematic review explores the association between environmental pollution – including PM2.5, PM10, NO_2_ and traffic emissions – and acne vulgaris. Of 27 identified studies, 17 met inclusion criteria, with several demonstrating links between pollutant exposure and acne exacerbation, potentially via oxidative stress and microbiome disruption. However, heterogeneity and limited adjustment for confounders highlight the need for more robust longitudinal research.

Acne vulgaris is a common and burdensome skin condition, particularly affecting adolescents and young adults. Emerging evidence suggests air pollution may contribute to the development or exacerbation of acne vulgaris. Environmental pollutants such as particulate matter (PM), nitrogen oxides (NO_x_) and polycyclic aromatic hydrocarbons (PAHs) have been shown to promote oxidative stress and inflammation which in turn can impair skin barrier function and potentially alter the cutaneous microbiome, all of which may be implicated in acne pathogenesis.^1^ While some studies have investigated the relationship between pollution and acne exacerbation, findings remain limited and often do not account for confounding variables. This systematic review aims to evaluate the current literature on the relationship between environmental pollution and acne outcomes.

## Materials and methods

### Study design

This systematic review was conducted following the PRISMA 2020 guidelines.^2^ The review protocol was registered in PROSPERO (CRD420251134623).

### Eligibility criteria

Studies were included if they met the following criteria:

- Population: humans of any age or sex.
- Exposure: environmental pollution, including but not limited to PM, NO_x_, sulfur dioxide, carbon monoxide and PAHs.
- Outcomes: acne vulgaris development, severity, sebum excretion rates (SERs), antioxidant levels, outpatient visits or acne-related symptoms.
- Study design: observational (cross-sectional, cohort, time-series), experimental or preclinical studies assessing pollutant exposure in relation to acne outcomes.
- Language: English-language studies.
- Time frame: studies published between 2010 and 2025.

Studies were excluded if they did not report acne outcomes or were case reports, conference abstracts or reviews lacking original data.

### Search strategy

A systematic search of PubMed was conducted to identify relevant peer-reviewed studies. The search strategy combined controlled vocabulary (Medical Subject Headings) terms and free-text keywords relating to environmental pollution and acne. Search terms included ‘air pollution’, ‘particulate matter’, ‘PM_2.5_’, ‘PM_10_’, ‘NO_2_’, ‘NO_x_’, ‘environmental pollution’, ‘traffic pollution’, ‘acne’ and ‘acne vulgaris’.

### Study selection

**Figure 1:**
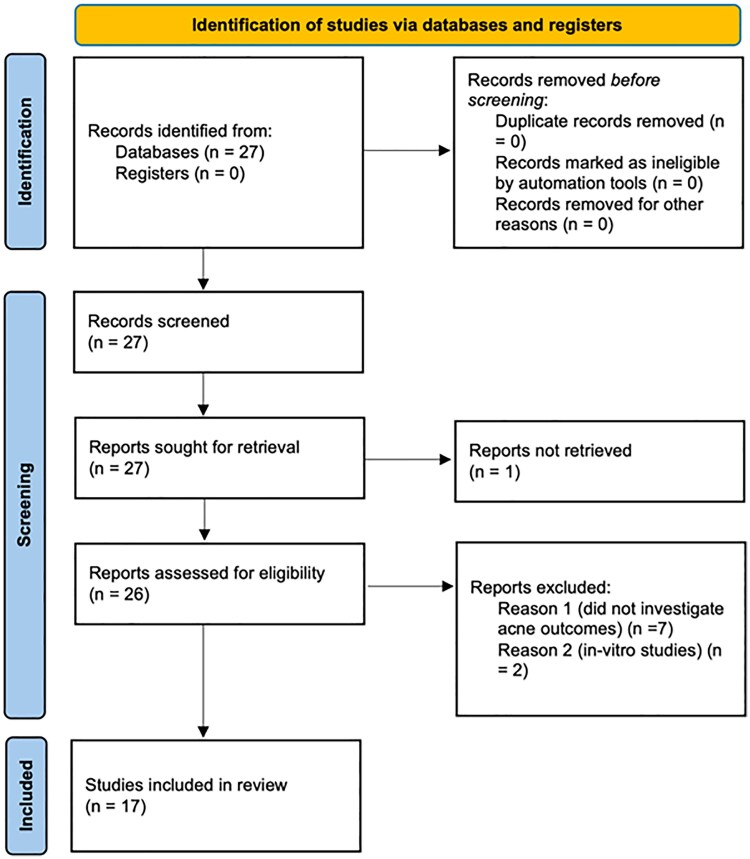
PRISMA flow diagram of the study.

Two independent reviewers screened titles and abstracts for eligibility, resolving any discrepancies through consensus with a third reviewer. A schematic presentation of the record selection process is outlined in the PRISMA flow diagram (Figure 1).

### Data extraction

Data were independently extracted by one reviewer and checked by another reviewer using a standardized form. Extracted information included:

- Study characteristics: author, year, country, study design, sample size, population characteristics.
- Exposure assessment: type and measurement of pollutants, exposure duration.
- Acne outcomes: severity measurements, clinical assessments, sebum excretion, antioxidant levels, outpatient visits.
- Key findings and effect estimates: odds ratios, relative risks or correlation coefficients, where available.

### Risk of bias assessment

Risk of bias was assessed independently by two reviewers using the Newcastle-Ottawa Scale. Disagreements were resolved by consensus.

### Data synthesis

Given the heterogeneity of study designs, pollutant measures and acne outcomes, a narrative synthesis was conducted. Key findings were summarized in tables and text according to pollutant type, exposure assessment method and acne outcome. Where feasible, trends across studies were highlighted and inconsistencies were discussed. Quantitative meta-analysis was not performed due to substantial methodological and outcome variability.

## Results

### Environmental pollutants

Recent attention has focused upon the adverse effects of environmental pollution upon health and disease states. Environmental pollutants are harmful substances present in the air, water and soil, which can be both naturally forming and anthropogenic. Commonly studied pollutants include PM, NO_x_ and PAHs, and many are monitored by national agencies. PM is classified by size: PM_2.5_ refers to fine particles with a diameter of ≤2.5 µm, while PM_10_ includes slightly larger particles up to 10 µm. These particles mainly emanate from traffic emissions, industrial activity and domestic fuel burning.^3^ NO_x_, comprising both nitrous oxide (NO) and nitrogen dioxide (NO_2_), are reactive gases predominantly released by power plants and vehicle exhausts, while PAHs are organic compounds generated during incomplete combustion of organic materials such as coal, oil and wood.^4^

Beyond their well-established roles in cardiovascular and respiratory diseases, including increased risk of heart disease, strokes and chronic lung conditions,^5^ these pollutants have also been implicated in various dermatological conditions.^6–11^

### Preclinical studies

Emerging evidence from preclinical and experimental studies suggest that pollutants may disrupt skin barrier function, increase oxidative stress (through generation of reactive oxygen species and depletion of antioxidants), promote inflammation, alter the cutaneous microbiome and stimulate sebum production – all mechanisms that plausibly contribute to acne development and exacerbation.^6–11^

A multi-omics observational study examined the effect of chronic exposure to pollution, gauged by PAH and metabolite levels in hair, upon clinical signs, including acne, pigmentation and wrinkles.^12^ This study demonstrated the PAH exposure was associated with alterations in the cutaneous microbiome and metabolic profiles, which could contribute to the development and exacerbation of acne by compromising skin barrier function.

A separate *in vitro* study simulated acne-like inflammation by stimulating neonatal human keratinocytes stimulated with *Cutibacterium acnes* or *Staphylococcus aureus* peptidoglycan.^13^ Whilst exposure to the bacteria induced inflammation, additional exposure to PM further increased expression of proinflammatory cytokines [interleukin (IL)‑1α, IL‑1β, IL‑6, IL‑8, tumour necrosis factor‑α], cyclooxygenase-2 protein, Toll-like receptor 4 and phosphorylation of nuclear factor‑κB. Taken together, these findings suggest that PM exposure may worsen acne by accentuated microbial-induced inflammation.

### Epidemiological association between air pollution and acne

While study designs and pollutant measurements varied, several studies support the association of exposure to ambient air pollution and poorer acne outcomes. Most studies employed the use of cross-sectional or longitudinal methods and utilized environmental monitoring data or ­satellite-derived estimates of pollutant exposure. Acne severity was typically assessed using dermatological grading scales or patient-reported outcomes. Table 1 summarizes all relevant studies investigating the association between air pollution and acne.

**Table 1:** Summary of studies investigating the association between environmental pollution and acne outcomes

Study	Sample size (*n*)	Study design	Country	Pollutants measured	Exposure measurement	Acne outcomes measurement	Summary of results
Chen **2024^14^**	4325	Cross-sectional study	China	PM_2.5_, PM_10_, O_3_, SO_2_, NO_2_, CO	AQMS	Machine learning model	Study investigated relationship between air pollution and skin sensitivity in patients with acne. This study revealed nonlinear relationships between secondary sensitive skin and PM (PM_2.5_, PM_10_), O_3_, SO_2_, NO_2_ and CO. The risk of secondary sensitive skin rises with PM_2.5_ concentrations, sharply increasing after 40 μg m^–3^. Fluctuations were observed with PM_10_ and NO_2_, while high SO_2_ levels significantly impacted the occurrence of sensitive skin. However, CO seemed to be a protective factor
Paik **2024^1^**	128 – across the population of 7 major Korean cities	Narrative review		PM		Overview of air pollution effects on skin	This narrative review comprises epidemiological studies investigating the association between PM and skin conditions. Four studies specifically examined the association between PM and acne. PM exposure was linked to increased acne outpatient visits. However, these results were not consistent amongst all four studies, with one cross-sectional study reporting no significant associations between PM and acne
Jin **2024^9^**		Narrative review		PAHs		Overview of air pollution effects on skin	In this study, the relationship between PAHs and acne was studied. PAHs were implicated in the development of chloracne via several proinflammatory pathways and increase in sebum production
Gu **2024^10^**		Narrative review		Various pollutants		Overview of air pollution effects on skin	Air pollution was implicated in the development of acne. Specifically, exposure to PAHs and O_3_ were linked to an increase in proinflammatory mediators which lead to the development of acne
Belzer **2023^11^**		Narrative review		PAHs, PM_2.5_, PM_10_, NO_2_		Overview of air pollution effects on skin	Air pollution exposure was linked with acne and other skin conditions such as atopic dermatitis, psoriasis and pigmentary disorders
Isler **2023^6^**		Narrative review		PAHs, NO_2_		Overview of air pollution effects on skin	This review demonstrated that exposure to pollutants such as PAHs and NO_2_ triggered changes to skin microbiome, which promotes the development of acne
Li **2022^15^**	120 842 outpatient visits	Time-series analysis	China	PM_2.5_	EMC	Acne outpatient visits	This study demonstrated positive associations between PM_2.5_ concentrations and acne outpatient visits. Specifically, a 10 μg m^–3^ increase in PM_2.5_ concentrations was associated with a 1.71% (95% CI 1.06–2.36) increase in acne outpatient visits over a 0–7-day lag period. Stratified analyses revealed that the effects of PM_2.5_ were more significant in individuals aged 25 years or over compared with those under 25 years, 2.26% (95% CI 1.37–3.15) and 1.24 (95% CI 0.38–2.10), respectively. However, there were no significant gender-based differences observed
Li **2022^16^**	71 625 outpatient visits	Time-series analysis	China	PM_10_, SO_2_, NO_2_	EMC	Acne outpatient visits	A 10 μg m^–3^ rise in SO_2_ and NO_2_ concentrations were linked to increased acne outpatient visits at lag zero, 1.02% (95% CI 0.16–1.88) and 2.13% (95% CI 0.98–3.28), respectively, with positive associations for both male and female participants. The effect of PM_10_ was statistically significant for adults over 30 years, with every 10 μg m^–3^ rise at lag 0–3 corresponding to 0.46% (95% CI 0.08–0.84%) increase of acne visits. However, no statistical difference was found between different gender and age subgroups when comparing SO_2_ and NO_2_
Misra **2021^12^**	134	Cross-sectional observational study	China	PAHs	AQMS	Dermatological clinical assessments	This study demonstrated that exposure to PAHs was associated with alterations in skin microbiota and metabolic profiles, which could compromise skin barrier functions and drive inflammation – changes contributing to the development and exacerbation of acne. However, direct causality between PAHs and acne was not established in this study
El Haddad **2021^17^**	372	Cross-sectional study	Lebanon	CO_2_, CO, SO_2_, NO_2_ and PM	AQMS	Patient-reported outcomes	Increased NO_2_ exposure was linked with a higher likelihood of developing acne (aOR 8.24). Working near a power plant also increased the risks of developing acne (aOR 3.07), as this was a source of pollutants, i.e. CO_2_, CO, SO_2_, NO_2_ and PM
Abolhasani **2021^18^**	22–252 799 outpatient visits	Narrative review		Various pollutants		Overview of air pollution effects on skin	This review included 103 studies. Many studies investigating the association between air pollution and acne reported significant associations with various pollutants and acne development and exacerbation. However, one Beijing study investigating the effect of air pollution on acne outpatient visits reported that increased SO_2_ levels were negatively associated with acne visits
Yang **2020^19^**	50–2 472 004	Systematic review		PM_2.5_, PM_10_, NO_2_		Patient-reported outcomes, acne prevalence	Review comprised 80 studies with a sample of 50–2 472 004 patients. Two studies comparing more polluted areas with less polluted areas revealed reduced skin quality correlated with continued exposure to ambient air pollution. A Beijing study demonstrated a positive correlation between increase in PM_2.5_, PM_10_ and NO_2_ concentrations and a rise in outpatient visits for acne vulgaris over the previous 2 years
Passeron **2020^20^**		Narrative review		PM_2.5_, PM_10_, NO_2_		Overview of air pollution effects on skin	This review demonstrated that there were positive associations between air pollution and the development of acne. A Mexican study included in this review (*n* = 189) compared the facial skin of people living in a polluted city and subjects living in a less polluted city. This study demonstrated qualitative differences and increased sebum production in patients who lived in the polluted city compared with patients in the less polluted city
Ali **2020^8^**		Narrative review		Various pollutants		Overview of air pollution effects on skin	This review demonstrated a relationship between pollutants and mechanisms which promote the development of acne such as inflammation, increased sebum production and oxidative stress
Dong **2019^21^**		Narrative review		PM_2.5_, PM_10_		Overview of air pollution effects on skin	This study demonstrated associations between PM and acne, with long term PM exposure being reported to aggravate inflammatory skin conditions such as acne, atopic dermatitis and psoriasis. PM exposure was also associated with the development of skin cancer
Liu **2018^22^**	59 325 outpatient visits	Time-series analysis	China	PM_2.5_, PM_10_, SO_2_, NO_2_	EMC	Acne outpatient visits	Increased concentrations of PM_2.5_, PM_10_ and NO_2_ were positively associated with increased acne outpatient visits over 2 years. However, an increase in SO_2_ concentrations showed negative correlation with acne outpatient visits in all test models used
Puri **2017^7^**		Narrative review		PAHs, PM, O_3_		Overview of air pollution effects on skin	Air pollution, particular PAH exposure was linked to the development of acneiform eruptions (chloracne), skin aging and pigmentation

aOR, adjusted odds ratio; AQMS, Air Quality Monitoring Stations; CI, confidence interval; CO, carbon monoxide; CO_2_, carbon dioxide; EMC, Environmental Monitoring Centre; NO_2_, nitrogen dioxide; O_3_, ozone; PAH, polycyclic aromatic hydrocarbon; PM, particulate matter (PM_2.5_, PM_10_); SO_2_, sulfur dioxide.

### Sebum excretion and antioxidant levels

A recent systematic review^19^ of 80 studies with sample sizes ranging from 50 to 2 472 004 participants, examined factors influencing acne development and severity. Of the 80 studies, 8 explored the relationship between acne and natural environmental factors, including air pollution. One of the cited clinical studies^23^ investigated the link between environmental pollution and acne by comparing 189 patients (96 from a highly polluted district of Mexico City and 93 from a less polluted district). Following clinical evaluation by dermatologists, it was found that patients from the less polluted district had a significantly lower SER compared with those from the more polluted district, with mean values of 64 and 100, respectively (*P* < 0.001). Additionally, a decrease in skin antioxidants was observed. Participants from the highly polluted district had significantly lower levels of both squalene (42.7 vs. 76.6; *P* < 0.001) and vitamin E (36.2 vs. 391.1; *P* < 0.001) compared with those from the less polluted district.

A complementary clinical study conducted in Shanghai^24^ investigated similar associations by comparing 159 participants – 79 patients with higher exposure to pollution and 80 patients with lower exposure. Industrialized countries such as China provide a valuable setting to investigate the relationship between air pollution and acne, given the consistently high concentration of PMs. However, unlike the Mexico City study, there was no statistically significant difference in SER between the groups, with mean values of 67 and 61 in the highly and less polluted areas, respectively. It is worth noting that SER values tend to be lower in Chinese populations when compared with Black and White American populations,^25^ which may partially explain this observation. Nonetheless, with regards to antioxidant levels, a similar trend to that observed in the Mexico City study was reported, with a significant decrease in squalene levels among those with higher pollution exposure (*P* < 0.05).

### Outpatient visits

A time-series study conducted in Xi’an China^16^ examining 71 625 outpatient visits for acne found that for every 10 μg m^–3^ rise in SO_2_ and NO_2_ concentrations, outpatient visits increased at lag 0 by 1.02% [95% confidence interval (CI) 0.16–1.88] and 2.13% (95% CI 0.98–3.28), respectively, with positive associations for both male and female participants. Here, ‘lag 0’ refers to the effects of exposure on the same day as the outpatient visit, whereas longer lag periods capture delayed or cumulative effects of exposure over several days. Interestingly, when comparing age subgroups, the effect of PM_10_ was statistically significant among adults over 30 years of age, with every 10 μg m^–3^ rise at lag 0–3 associated with a 0.46% (95% CI 0.08–0.84%) increase in acne visits. However, no significant trends were observed across age groups for SO_2_ and NO_2_.

Another time-series study in Chongqing, China^15^ investigating the relationship between PM_2.5_ exposure and acne outpatient visits (*n* = 120 842) demonstrated a positive association between higher PM_2.5_ concentrations and increased outpatient visits. Specifically, a 10 μg m^–3^ rise in PM_2.5_ concentrations was associated with a 1.71% increase in acne outpatient visits over a 0- to 7-day lag period (95% CI 1.06–2.36). Stratified analyses revealed that the impact of PM_2.5_ was more pronounced in individuals over 25 years (2.26%; 95% CI 1.37–3.15) compared with those under 25 years of age (1.24%; 95% CI 0.38–2.10). However, no significant gender-based differences were observed.

A third time-series analysis,^22^ also examining acne outpatient visits in Beijing, China (*n* = 59 325), reported similar findings, with higher PM_10_, PM_2.5_ and NO_2_ concentrations being positively associated with increased outpatient visits. Notably, increases in SO_2_ concentrations appeared to have a protective effect, showing a negative correlation with acne outpatient visits across all test models.

### Acne symptoms

A cross-sectional study across several regions in China investigated the relationship between air pollution and skin sensitivity secondary to acne (assessed by questionnaire).^14^ This study involved 4325 patients with acne and revealed nonlinear relationships between secondary sensitive skin and PM_2.5_, PM_10_, SO_2_, NO_2_ and carbon monoxide (CO). The risk of developing secondary sensitive skin increased with rising PM_2.5_ concentrations, with a sharp escalation observed above 40 μg m^–3^. Elevated SO_2_ levels also significantly impacted the occurrence of sensitive skin, while PM_10_ and NO_2_ showed fluctuating effects. Interestingly, CO seemed to be protective in this study.

A cross-sectional study involving 372 participants in Lebanon investigated the associations between pollutant exposure and the risk of developing acne.^17^ Increased exposure to NO_2_ was significantly associated with a higher likelihood of developing acne [adjusted odds ratio (aOR) 8.24]. Additionally, participants working near a power plant – identified as a source of CO, SO_2_, NO_2_ and PM – also showed an elevated risk of acne (aOR 3.07).

Overall, the evidence from the epidemiological studies indicates a potential link between increased exposure to air pollution and increased acne prevalence or severity. While findings report minor inconsistencies, likely due to differences in population demographics, study types and pollutant types, most studies support an association between exposure to pollutants such as PM_2.5_, NO_2_ and SO_2_ and negative acne-related outcomes.

## Discussion

This systematic review synthesizes emerging evidence suggesting a positive association between environmental pollution and acne outcomes. Multiple epidemiological studies, particularly large-scale time-series studies conducted in China, consistently demonstrated that higher concentrations of airborne pollutants – especially PM_2.5_, PM_10_, NO_2_ and SO_2_ – were correlated with increased outpatient visits for acne. These findings are supported by observational and cross-sectional studies from diverse settings, including Mexico and Lebanon, further reinforcing the potential impact of pollutant exposure on acne severity, SER and skin antioxidant levels.

Mechanistic pathways proposed by some studies provide plausible biological explanations for these associations. Pollutants such as PM and NO_2_ may exacerbate acne through oxidative stress, inflammation, and disruption of the skin barrier and microbiome. The observational studies discussed suggest that increased pollution may be linked to higher SER and reduced antioxidant levels (vitamin E and squalene). This is particularly relevant, as these factors promote comedogenicity, oxidative stress and skin dysbiosis – all of which are key contributors to acne pathogenesis.^26^

It is important to note that, while most studies showed positive associations, a few reported nonsignificant or protective effects, particularly with pollutants like CO or SO_2_ in certain models. These findings may reflect real biological variation or methodological inconsistencies, warranting further investigations.

We acknowledge that limitations in the literature to date, namely heterogeneity in study design, pollutant measurement methods and acne outcomes assessments, limited comparability. Confounding variables such as individual pollutant exposure, diet, skincare practices and socioeconomic factors were often not accounted for. There was a paucity of geographic and ethnic diversity, which may limit the generalizability of findings, with most studies conducted in East Asia, with limited data from Western, African or other global populations. Cross-sectional designs make up a vast majority of the literature, limiting causal inferences. Longitudinal and interventional studies remain scarce.

This review underscores the need for future high-quality research to better define the causal relationship between air pollution and acne. Longitudinal studies with standardized pollution exposure assessment, acne outcome assessments and control for key confounders are crucial. Moreover, studies exploring vulnerable subgroups such as adolescents, individuals with pre-existing skin conditions and people living in heavily industrialized areas are needed to identify populations at greatest risk. Beyond acne, air pollution has also been implicated in the pathogenesis and exacerbation of other dermatological conditions including atopic dermatitis, skin ageing and psoriasis.^27–29^ Given this, future research could benefit from a broader dermatological perspective to fully grasp its impact on cutaneous health.

### Implications for dermatologists

Recognition of air pollution as a potentially modifiable environmental risk factor for acne may help inform holistic patient care and urban health policy. For patients with recalcitrant acne, a brief environmental exposure history (e.g. proximity to heavy traffic or industrial areas) may be relevant. The established links between occupational exposures and acne – such as polyhalogenated hydrocarbons causing chloracne,^7,9^ and coal tar or petrol derivatives contributing to occupational acne^30^ – serve as a reminder that environmental factors can play a role in acne ­pathogenesis.

While the precise mechanisms by which common urban pollutants worsen acne remain incompletely understood, dermatologists may reasonably offer pragmatic skincare advice for patients in high-exposure settings (e.g. cyclists, roadside workers and outdoor labourers). These include twice-daily cleansing to putatively remove surface residues, use of a light noncomedogenic moisturizer to support the skin barrier, broad-spectrum sunscreen and considering antioxidant-containing products to offset some of oxidative stress induced by pollution.

## Conclusion

This systematic review highlights a growing body of evidence supporting a link between environmental pollution and the development or exacerbation of acne. Although the findings are not entirely consistent across all studies, air pollution may act as an environmental trigger or aggravator of acne through oxidative stress, inflammation, and disruption of the skin barrier and microbiome.

However, limitations in study design, geographic scope and confounder control reduce the strength of current conclusions. As urbanization and environmental pollution continue to rise globally, it is increasingly important to understand how these exposures impact common skin conditions such as acne. Further high-quality, longitudinal and mechanistic research is needed to establish causality, identify high-risk groups and guide preventative ­strategies.

Incorporating environmental factors into dermatological assessments may support more personalized and comprehensive acne management. Ultimately, acknowledging pollution as a potential contributor to acne aligns with broader public health goals aimed at reducing environmental harm and promoting skin health.

### Author contributions

Isobel R. Okeah (Methodology [lead], Writing—original draft [lead], Writing—review & editing [equal]), Usamah M. Afzal (Resources [equal], Supervision [supporting], Writing—review & editing [equal]) and Faisal R. Ali (Conceptualization [lead], Resources [equal], Supervision [lead], Writing—review & editing [equal])

## Data availability

No new data were generated or analysed in support of this research.

## References

[1] Paik K, Na JI, Huh CH et al Particulate matter and its molecular effects on skin: implications for various skin diseases. Int J Mol Sci 2024; 25:9888. 39337376 10.3390/ijms25189888 PMC11432173

[2] Page MJ, McKenzie JE, Bossuyt PM et al The PRISMA 2020 statement: an updated guideline for reporting systematic reviews. BMJ 2021; 372:n71. 33782057 10.1136/bmj.n71 PMC8005924

[3] Buiarelli F, Di Filippo P, Massimi L et al Ultrafine, fine and coarse airborne particle mass concentration in workplaces. Atmos Pollut Res 2019; 10:1685–90.

[4] Goshua A, Akdis CA, Nadeau KC. World Health Organization global air quality guideline recommendations: executive summary. Allergy 2022; 77:1955–60. 35060140 10.1111/all.15224 PMC12052406

[5] Kampa M, Castanas E. Human health effects of air pollution. Environmental Pollution 2008; 151:362–7. 17646040 10.1016/j.envpol.2007.06.012

[6] Isler MF, Coates SJ, Boos MD. Climate change, the cutaneous microbiome and skin disease: implications for a warming world. Int J Dermatol 2023; 62:337–45. 35599301 10.1111/ijd.16297

[7] Puri P, Nandar S, Kathuria S et al Effects of air pollution on the skin: a review. Indian J Dermatol Venereol Leprol 2017; 83:415. 28195077 10.4103/0378-6323.199579

[8] Ali A, Khan H, Bahadar R et al The impact of airborne pollution and exposure to solar ultraviolet radiation on skin: mechanistic and physiological insight. Environ Sci Pollut Res Int 2020; 27:28730–6. 32462622 10.1007/s11356-020-09280-4

[9] Jin H, Lin Z, Pang T et al Effects and mechanisms of polycyclic aromatic hydrocarbons in inflammatory skin diseases. Sci Total Environ 2024; 925:171492. 38458465 10.1016/j.scitotenv.2024.171492

[10] Gu X, Li Z, Su J. Air pollution and skin diseases: a comprehensive evaluation of the associated mechanism. Ecotoxicol Environ Saf 2024; 278:116429. 38718731 10.1016/j.ecoenv.2024.116429

[11] Belzer A, Parker ER. Climate change, skin health, and dermatologic disease: a guide for the dermatologist. Am J Clin Dermatol 2023; 24:577–93. 37336870 10.1007/s40257-023-00770-y

[12] Misra N, Clavaud C, Guinot F et al Multi-omics analysis to decipher the molecular link between chronic exposure to pollution and human skin dysfunction. Sci Rep 2021; 11:18302. 34526566 10.1038/s41598-021-97572-1 PMC8443591

[13] Noh HH, Shin SH, Roh YJ et al Particulate matter increases Cutibacterium acnes-induced inflammation in human epidermal keratinocytes via the TLR4/NF-κB pathway. PLoS One 2022; 17:e0268595. 35947554 10.1371/journal.pone.0268595 PMC9365135

[14] Chen X, Wen J, Wu W et al Non-linear association between air pollutants and secondary sensitive skin in acne patients. J Cosmet Dermatol 2024; 23:4007–16. 39057602 10.1111/jocd.16487 PMC11626370

[15] Li X, Zhou LX, Yang LL et al The relationship between short-term PM2.5 exposure and outpatient visits for acne vulgaris in Chongqing, China: a time-series study. Environ Sci Pollut Res Int 2022; 29:61502–11. 35442002 10.1007/s11356-022-20236-8

[16] Li X, Cao Y, An SJ et al The association between short-term ambient air pollution and acne vulgaris outpatient visits: a hospital-based time-series analysis in Xi’an. Environ Sci Pollut Res Int 2022; 29:14624–33. 34617215 10.1007/s11356-021-16607-2

[17] El Haddad C, Gerbaka NE, Hallit S et al Association between exposure to ambient air pollution and occurrence of inflammatory acne in the adult population. BMC Public Health 2021; 21:1664. 34521361 10.1186/s12889-021-11738-0 PMC8439009

[18] Abolhasani R, Araghi F, Tabary M et al The impact of air pollution on skin and related disorders: a comprehensive review. Dermatol Ther 2021; 34:e14840. 33527709 10.1111/dth.14840

[19] Yang J, Yang H, Xu A et al A review of advancement on influencing factors of acne: an emphasis on environment characteristics. Front Public Health 2020; 8:450. 33042936 10.3389/fpubh.2020.00450 PMC7527424

[20] Passeron T, Krutmann J, Andersen ML et al Clinical and biological impact of the exposome on the skin. J Eur Acad Dermatol Venereol 2020; 34:4–25. 10.1111/jdv.16614 32677068

[21] Dong YM, Liao LY, Li L et al Skin inflammation induced by ambient particulate matter in China. Sci Total Environ 2019; 682:364–73. 31125750 10.1016/j.scitotenv.2019.05.155

[22] Liu W, Pan X, Vierkötter A et al A time-series study of the effect of air pollution on outpatient visits for acne vulgaris in Beijing. Skin Pharmacol Physiol 2018; 31:107–13. 29408821 10.1159/000484482

[23] Lefebvre M-A, Pham D-M, Boussouira B et al Evaluation of the impact of urban pollution on the quality of skin: a multicentre study in Mexico. Int J Cosmet Sci 2015; 37:329–38. 25655908 10.1111/ics.12203

[24] Lefebvre M, Pham D, Boussouira B et al Consequences of urban pollution upon skin status. A controlled study in Shanghai area. Int J Cosmet Sci 2016; 38:217–23. 26291783 10.1111/ics.12270

[25] Nouveau-Richard S, Zhu W, Li YH et al Oily skin: specific features in Chinese women. Skin Res Technol 2007; 13:43–8. 17250531 10.1111/j.1600-0846.2006.00185.x

[26] Del Rosso JQ, Kircik L. The primary role of sebum in the pathophysiology of acne vulgaris and its therapeutic relevance in acne management. J Dermatolog Treat 2024; 35:2296855. 38146664 10.1080/09546634.2023.2296855

[27] Fadadu RP, Chee E, Jung A et al Air pollution and global healthcare use for atopic dermatitis: a systematic review. J Eur Acad Dermatol Venereol 2023; 37:1958–70. 37184289 10.1111/jdv.19193

[28] Robic J, Lata W, Nkengne A et al The impact of air pollution on the facial skin of Caucasian women using real-life pollutant exposure measurements. Skin Res Technol 2024; 30:e13669. 38965805 10.1111/srt.13669 PMC11224121

[29] Wu J, Ma Y, Yang J et al Exposure to air pollution, genetic susceptibility, and psoriasis risk in the UK. JAMA Netw Open 2024; 7:e2421665. 39012635 10.1001/jamanetworkopen.2024.21665 PMC11252902

[30] Demir B, Cicek D. Occupational acne. In: Kartal SP, Gonul M (eds). Acne and Acneiform Eruptions. Rijeka (Croatia): InTech, 2017, 53–67.

